# Patient satisfaction and pain relief following radiofrequency rhizotomy for trigeminal neuralgia: a prospective cohort study

**DOI:** 10.3389/fpain.2025.1639140

**Published:** 2025-09-01

**Authors:** Eyad Faizo, Maher Kurdi, Badr Hafiz, Wareef Alzahrani, Norah Alajmi, Bashayer Althaqafi, Raed Gasemaltayeb, Afaf Albalawi, Ahmad A. Fallata, Iman Mirza, Ahmed Najjar, Mohammed Alyousef, Alaa Alkhotani, Saleh Baeesa

**Affiliations:** ^1^ Department of Surgery, Faculty of Medicine, University of Tabuk, Tabuk, Saudi Arabia; ^2^ Department of Surgery, Doctor Soliman Fakeeh Hospital, Jeddah, Saudi Arabia; ^3^ Department of Pathology, Faculty of Medicine, King Abdulaziz University, Rabigh, Saudi Arabia; ^4^ Department of Neurosciences, King Faisal Specialist Hospital and Research Center, Jeddah, Saudi Arabia; ^5^ Department of Neurosurgery, National Guard Hospital, Jeddah, Saudi Arabia; ^6^ Department of Neurosurgery, Prince Sultan Military Medical City, Riyadh, Saudi Arabia; ^7^ Department of Neurosurgery, Anoor Specialist Hospital, Mecca, Saudi Arabia; ^8^ Department of Internal Medicine, Faculty of Medicine, King Abdulaziz University, Rabigh, Saudi Arabia; ^9^ Department of Internal Medicine, Faculty of Medicine, University of Tabuk, Tabuk, Saudi Arabia; ^10^ Department of Family Medicine, Faculty of Medicine, University of Tabuk, Tabuk, Saudi Arabia; ^11^ Department of General and Specialized Surgery, College of Medicine, Taibah University, Madinah, Saudi Arabia; ^12^ Department of Surgery, Faculty of Medicine, King Abdulaziz University, Jeddah, Saudi Arabia; ^13^ Department of Pathology, College of Medicine, Umm Al-Qura University, Mecca, Saudi Arabia

**Keywords:** trigeminal neuralgia, radiofrequency rhizotomy, pain management, patient satisfaction, patient reported outcomes, prospective study

## Abstract

**Background:**

Trigeminal neuralgia (TN) causes severe facial pain and affects quality of life. Radiofrequency rhizotomy (RFR) is often used when medications fail. This multicenter study assesses pain relief and patient satisfaction following this minimally invasive procedure in affected individuals treated across multiple institutions in Saudi Arabia and Pakistan.

**Methods:**

In this prospective cohort study, 50 patients aged 40–60 with medically refractory TN (V2/V3) underwent percutaneous RFR at 75°C for 60 s under fluoroscopy, followed by dexamethasone injection. Pain (VAS) and patient satisfaction (PGIC) were evaluated at 1 and 6 months post-procedure. Inter-statistical analysis of patients' clinical outcomes using repeated measures ANOVA and Chi-square tests was performed.

**Results:**

The average age of the participants was 50.58 ± 5.67 years. 72% were female. The right side was more commonly affected (62%) than the left (38%). The maxillary branch (V2) was the most frequently involved (76%), followed by the mandibular branch (V3) in 24%. Pain scores significantly decreased from a baseline mean of 8.04 ± 0.78–3.20 ±  1.05 at 1 month and 2.58 ± 1.18 at 6 months (p  <  0.001). Patient satisfaction scores also improved significantly, from 5.60 ± 1.20 at baseline to 2.52 ± 1.11 at 1 month and 1.92 ± 0.80 at 6 months (p < 0.001). The most common complication was facial numbness (32%), followed by masticator weakness (14%), dysesthesia (6%), hematoma (2%) and pain recurrence occurred in 6% of cases, defined by either an increase in VAS ≥ 4 or the need for a second intervention during the follow-up window.

**Conclusion:**

Radiofrequency rhizotomy offers effective, well-tolerated pain relief for trigeminal neuralgia with high patient satisfaction. It improves symptom control and outcomes, though further long-term studies are needed to assess sustained benefits and quality-of-life impacts beyond six months.

## Introduction

Trigeminal neuralgia (TN) is a chronic neuropathic condition characterised by recurrent episodes of facial pain arising from dysfunction of the trigeminal nerve (1, 2). The condition affects an estimated 12 individuals per 100,000 annually, with significant variability in clinical presentation. Two primary subtypes are recognised: Type I, marked by sudden, shock-like pain attacks, and Type II, which involves a more constant ache interspersed with exacerbations of sharp pain (3, 4). The natural history of TN also varies considerably. At the same time, some patients experience spontaneous remissions, and many report gradual progression, which may extend to additional branches of the nerve or even affect the opposite side of the face (5). Although the precise mechanisms underlying TN remain unclear, current evidence suggests that neurovascular compression at the nerve root entry zone plays a key role in many cases. Initial management typically involves pharmacological therapy, with agents such as carbamazepine and oxcarbazepine being commonly prescribed (6). However, as the condition advances, medications often lose efficacy or produce intolerable side effects, prompting consideration of procedural options (5, 6).

Percutaneous radiofrequency rhizotomy (RFR) has emerged as a well-established, minimally invasive intervention for drug-resistant TN (7, 8). This procedure involves the application of controlled thermal energy via an electrode inserted through the foramen ovale, selectively disrupting nociceptive fibres to reduce pain perception, a process known as thermal neurolysis (9). While RFR can offer substantial pain relief, recurrence is not uncommon, and many patients eventually require repeat treatments. Reports suggest that approximately half of treated individuals may experience recurrence within five years (10–12).

Despite the long-standing use of RFR, there remains no clear consensus on the ideal procedural settings, particularly concerning the optimal ablation temperature and duration. Current practices often rely on institutional protocols or individual surgeon preference, emphasising the need for evidence-based guidance. Compared to alternatives like microvascular decompression (MVD), which remains the gold standard for medically refractory cases, RFR offers a more straightforward and less invasive solution. Recent reviews report durable pain relief in over 80% of MVD patients with recurrence rates of 10%–15% at 2–5 years (11, 12). However, MVD may not be feasible or acceptable for elderly patients or those with comorbidities, making RFR a valuable and accessible option.

Previous prospective studies have compared different temperature thresholds, such as 62°C to 75°C, with some suggesting improved long-term outcomes at higher settings. However, most of these studies maintained fixed ablation durations and did not explore the impact of varying time intervals (13). Compared to alternatives like microvascular decompression (MVD) or peripheral neurectomy, RFR offers effective and targeted ablation with relatively lower procedural complexity and fewer systemic risks (14). However, although earlier studies such as those by Nanjappa et al. and Cheng et al. have highlighted the effectiveness of RFR in managing pain, limited research has focused on patient satisfaction and post-treatment quality of life (15). Moreover, treatments such as endoscopic MVD or ganglion-based interventions are often less accessible in resource-constrained settings, underscoring the practical value of RFR (6, 16). This study, conducted across several tertiary centres, aims to assess both pain reduction and patient-reported satisfaction following percutaneous radiofrequency ablation in patients with pharmacologically refractory TN, using validated clinical outcome metrics over a six-month follow-up.

## Materials and methods

This multicenter prospective cohort study was conducted across three Institutions: the University of Lahore (Pakistan), the University of Tabuk, and the King Abdulaziz Medical City (Saudi Arabia) between April and December 2024, following ethical approval from the institutional review board. A total of 50 patients were enrolled, with the sample size determined using an anticipated 97% pain relief rate after RFR, a 5% margin of error, 5% significance level, and an expected 10% dropout rate. Patients aged 40–60 years were included to control for confounding variables such as age-related neural degeneration, comorbidities, and increased procedural risk outside this range, and TN involving the V2 and/or V3 branches was eligible. Medically refractory TN was defined as inadequate pain control despite at least 600 mg/day of carbamazepine for ≥6 weeks or documented intolerance to first-line agents (e.g., dizziness, rash, hyponatremia). Both classical Type I and Type II TN subtypes were eligible. None of the patients in this cohort had undergone prior rhizotomy, ensuring procedural uniformity and unbiased evaluation of primary outcomes. Exclusion criteria included local infection at the puncture site, V1 involvement, bleeding disorders, psychiatric illness, and uncontrolled hypertension.

### Treatment procedure

Patients were placed supine with neck extension for C-arm fluoroscopic access. The Hartel's approach was used to introduce a 22-gauge radiofrequency cannula through the foramen ovale. Sensory stimulation at 0.2–0.4 V reproduced paresthesia in the affected branch; motor stimulation was limited to <0.5 V to avoid excessive masseter contraction. A single thermal lesion was applied at 75°C for 60 s, with the cannula tip positioned adjacent to the trigeminal ganglion. Dexamethasone (0.5 ml of 4 mg/ml) was injected through the cannula to reduce periganglionic inflammation and minimise neuritis-related complications, as previously supported in procedural pain literature (17, 18).

### Outcome assessment

Pain intensity was evaluated using the Visual Analogue Scale (VAS). At the same time, patient satisfaction was assessed via the Patient Global Impression of Change (PGIC), a validated single-item measure reflecting perceived improvement post-treatment. As the PGIC is validated solely as a post-intervention global rating, it was administered only at follow-up visits (1 and 6 months), not at baseline. The PGIC employs a 7-point Likert scale, with scores from 1 (very much improved) to 7 (very much worse). Scores of 1–3 signify improvement, 4 indicates no change, and 5–7 denote worsening. Patients scoring between 1 and 3 were considered satisfied with the treatment outcome (19, 20).

### Follow-up plan

Treatment was considered adequate if the patient had a VAS score of less than 3 and rated their satisfaction as improved. A VAS score <3 It is widely used in neurosurgical literature to reflect mild or tolerable pain levels unlikely to interfere with activities of daily living, thus serving as a clinically relevant benchmark for treatment success (15, 16, 21). Recurrence was defined as an increase in VAS score to ≥4 following initial pain relief, or the requirement of a repeat RFR procedure within the follow-up period. Outcomes indicating no change or deterioration were classified as unsuccessful. Follow-up evaluations were conducted at baseline, 1 month, and 6 months after the procedure, encompassing VAS scores, patient satisfaction levels, and any complications related to the intervention.

## Statistical analysis

Quantitative data such as age and VAS scores were expressed as means ± standard deviation, while categorical variables like gender, nerve involvement, and site were presented as frequencies and percentages. Repeated measures ANOVA was applied to evaluate changes in pain (VAS) and satisfaction (PGIC) scores over time (baseline, 1 month, and 6 months), as this method accounts for within-subject correlations. *Post hoc* Least Significant Difference (LSD) tests were used for pairwise comparisons between time points. Data were stratified by gender, nerve branch, and site, and associations with treatment outcomes were examined using the Chi-square test. A *p*-value < 0.05 indicated significance. The Chi-square test was used to investigate associations between static categorical variables (e.g., gender, nerve branch, side) and overall treatment success at 6 months. McNemar's test was not applicable, as the variables under analysis were not repeated measures. Analyses were performed using SPSS version 25.

## Results

### Baseline features of TN patients undergoing RRF

The average age of the participants was 50.58 ± 5.67 years. Among the 50 patients included in the study, the majority were female (72%), while males accounted for 28%. The right side of the face was more frequently affected (62%) compared to the left side (38%) (Table 1). Regarding the distribution of trigeminal nerve branches involved, the maxillary branch (V2) was the most affected (76%), followed by the mandibular branch (V3) in 24% of cases.

**Table 1:** Baseline characteristics of patients with trigeminal neuralgia undergoing radiofrequency rhizotomy.

Variable	Frequency (%)
Age [Median(IQR)]	50 (10.25)
Gender	
Female	36 (72.0%)
Male	14 (28.0%)
Site involved	
Right	31 (62.0%)
Left	19 (3.0%)
Affected Nerve branches	
V2	38 (76.0%)
V3	12 (24.0%)

Additionally, among the 18 patients who had reached at least 9 months post-procedure by the time of manuscript revision, 14 (77.8%) continued to report low pain scores (VAS < 3) and sustained satisfaction (PGIC scores 1–3). Three patients experienced partial recurrence requiring adjustment in analgesic therapy, and one patient underwent repeat RFR. While limited by partial follow-up, these data provide early support for the sustained efficacy of RFR beyond the six-month mark.

### Pain and satisfaction scores at baseline, 1 month, and 6 months after RFR

**Figure 1:**
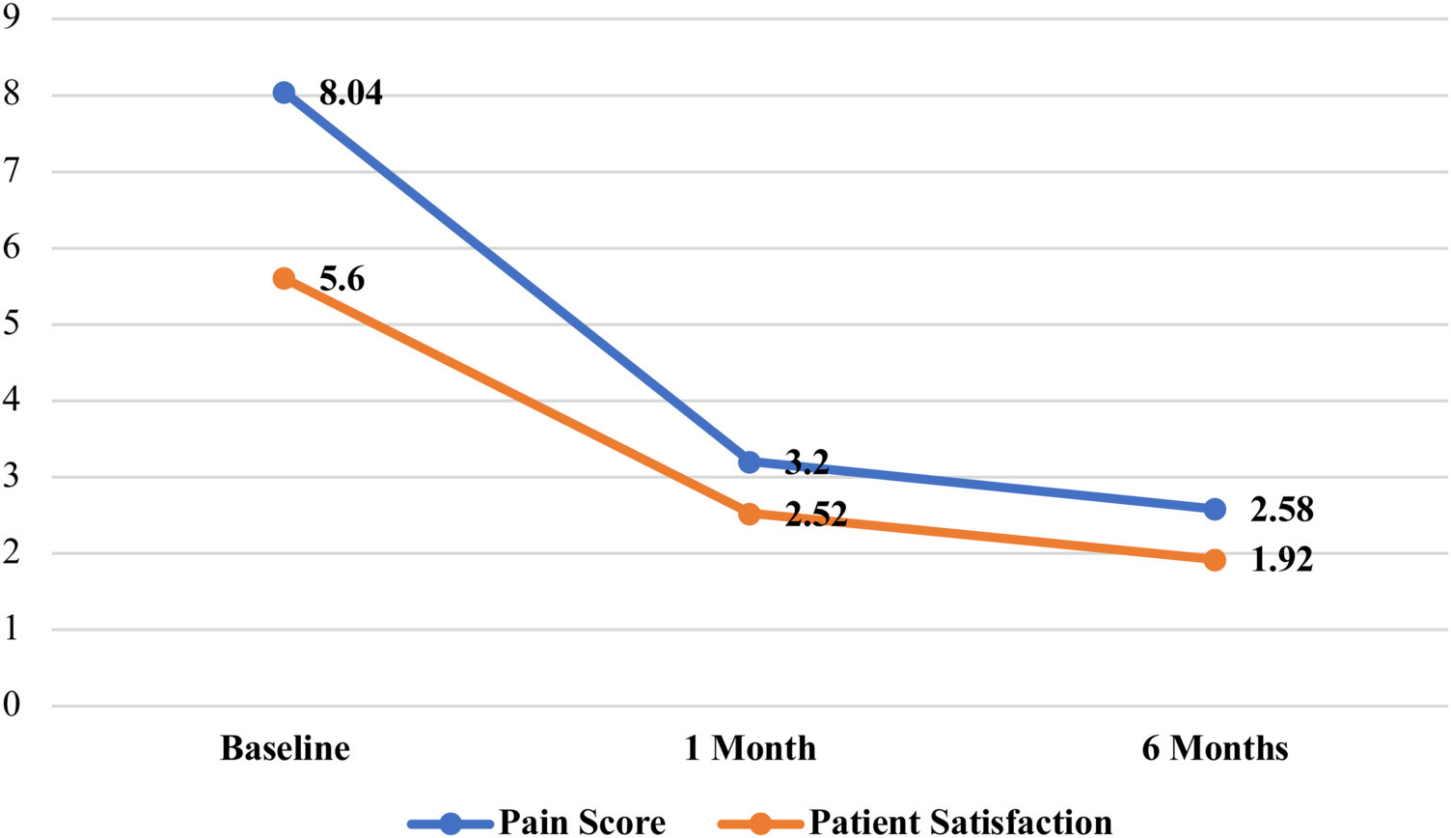
Trends in pain score and patient satisfaction over time following RFR.

The mean baseline pain score was 8.04 ± 0.78, which showed a significant reduction to 3.20 ± 1.05 at 1 month and further declined to 2.58 ± 1.18 at 6 months following the procedure (*p* < 0.001) (Table 2) (Figure 1). Similarly, patient satisfaction has improved over time, with a mean score of 5.60 ± 1.20 at baseline, decreasing to 2.52 ± 1.11 at 1 month and 1.92 ± 0.80 at 6 months, showing a statistically significant difference (*p* < 0.001) (Table 2).

**Table 2:** Pain and patient satisfaction scores at baseline, 1 month, and 6 months following radiofrequency rhizotomy in patients with trigeminal neuralgia (*n* = 50).

Variable	Time Point	Mean ± SD	95% CI (Lower–Upper)	*p*-value
Pain Score	Baseline	8.04 ± 0.78	7.82–8.26	<0.001
	1 Month	3.20 ± 1.05	2.90–3.50	
	6 Months	2.58 ± 1.18	2.24–2.92	
Patient Satisfaction	Baseline	5.60 ± 1.20	5.26–5.94	<0.001
	1 Month	2.52 ± 1.11	2.20–2.84	
	6 Months	1.92 ± 0.80	1.69–2.15	

### Multiple comparisons (LSD) for pain score and patient satisfaction

Repeated measures ANOVA demonstrated significant overall differences across time points for both pain and satisfaction scores (*p* < 0.001 for both variables). The pairwise comparisons revealed statistically significant reductions in both pain scores and patient satisfaction scores over time following RFR. There was a substantial decrease in mean pain score from baseline to 1 month (Mean Difference = 4.84, *p* < 0.001) and from baseline to 6 months (Mean Difference = 5.46, *p* < 0.001) (Table 3). Additionally, a more minor but statistically significant reduction was observed between 1 month and 6 months (Mean Difference = 0.62, *p* = 0.003), indicating continued improvement. Satisfaction significantly improved from baseline to 1 month (Mean Difference = 3.08, *p* < 0.001) and further by 6 months (Mean Difference = 3.68, *p* < 0.001). The increase in satisfaction between 1 month and 6 months was also significant (Mean Difference = 0.60, *p* = 0.005) (Table 3).

**Table 3:** *Post hoc* pairwise comparisons (LSD) following repeated measures ANOVA for pain score and patient satisfaction.

Dependent Variable	Time Comparison	Mean Difference (I–J)	*p*-value	95% Confidence Interval
Pain Score	Baseline vs. 1 Month	4.84	<0.001	4.44–5.24
	Baseline vs. 6 Months	5.46	<0.001	5.06–5.86
	1 Month vs. 6 Months	0.62	0.003	0.22–1.02
Patient Satisfaction	Baseline vs. 1 Month	3.08	<0.001	2.66–3.50
	Baseline vs. 6 Months	3.68	<0.001	3.26–4.10
	1 Month vs. 6 Months	0.60	0.005	0.18–1.02

### Postoperative complications after RFR

**Figure 2:**
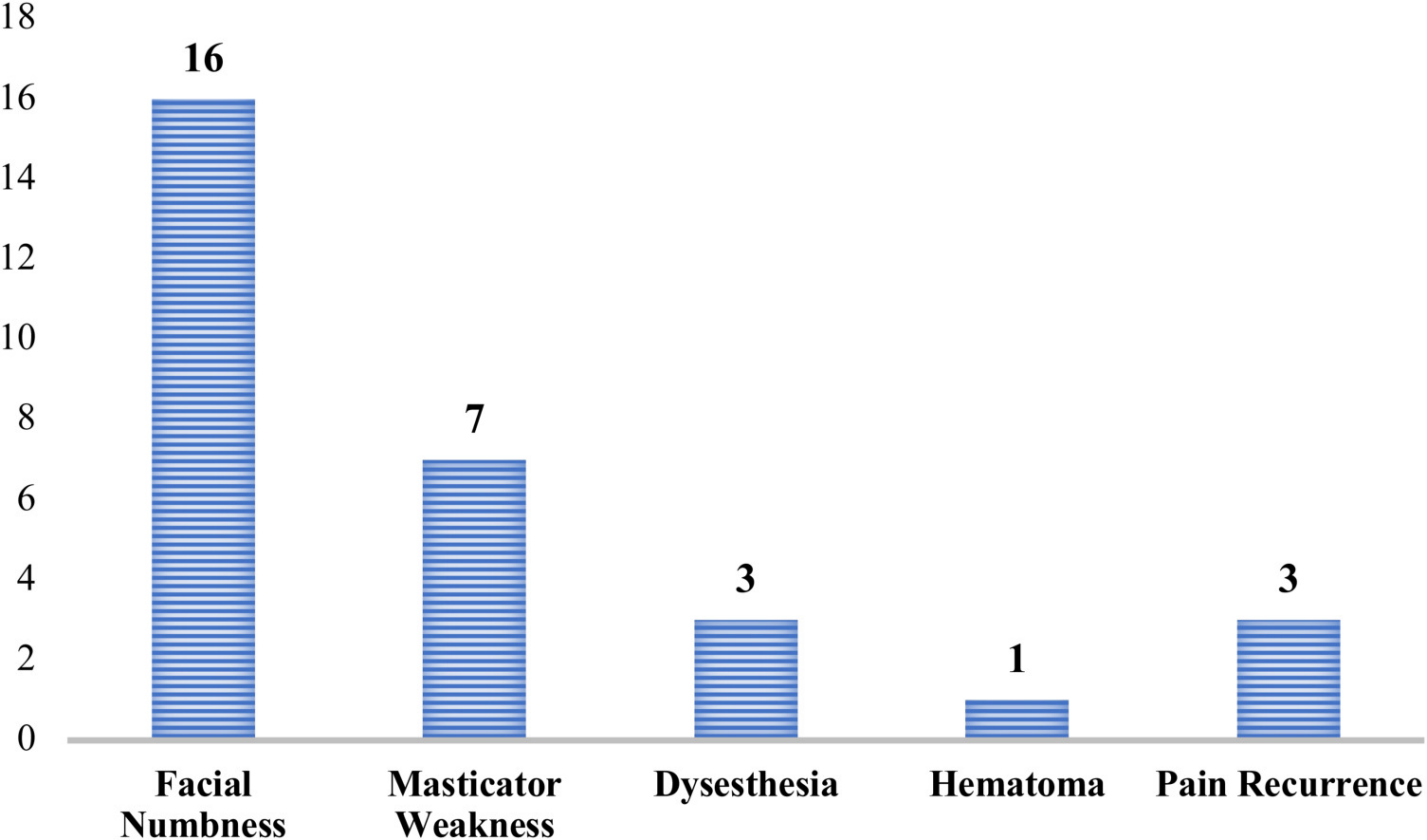
Frequency of postoperative complications following RFR for TN.

Among the patients undergoing RFR, the most frequently reported complication was facial numbness, observed in 16 patients (32%) (Figure 2). This was followed by masticator weakness in 7 patients (14%), and dysesthesia and pain recurrence occurred in 6% of cases, defined by either an increase in VAS ≥4 or the need for a second intervention during the follow-up window. A single case (2%) of hematoma was reported. A subgroup analysis comparing PGIC scores in patients with vs. without postoperative complications (e.g., facial numbness or masticator weakness) revealed no statistically significant differences at 6 months (*p* = 0.218). However, a mild trend toward reduced satisfaction was observed among those with persistent numbness.

### The associations between demographic and clinical variables and treatment outcomes

Chi-square analysis was performed to examine the associations between demographic and clinical variables and treatment outcomes (success vs. failure). Although female patients showed a higher rate of treatment success (74.4%) compared to males (25.6%), the association between gender and outcome was not statistically significant (*χ*² = 1.01, *p* = 0.315) (Table 4). Similarly, the site of involvement (right vs. left) and affected nerve branch (V2 vs. V3; V1 cases were excluded) did not demonstrate significant associations with treatment outcome (site: *χ*² = 1.37, *p* = 0.241; nerve branch: *χ*² = 0.52, *p* = 0.470).

**Table 4:** Chi-square analysis of associations between demographic and clinical variables and treatment outcomes (success vs. failure).

Variable	Category	Success (*n* = 43)	Failure (*n* = 7)	Total (*n* = 50)	*χ*² (df)	*p*-value
Gender	Female	32 (74.4%)	4 (57.1%)	36 (72.0%)	1.01 (1)	0.315
	Male	11 (25.6%)	3 (42.9%)	14 (28.0%)		
Site Involved	Right	28 (65.1%)	3 (42.9%)	31 (62.0%)	1.37 (1)	0.241
	Left	15 (34.9%)	4 (57.1%)	19 (38.0%)		
Affected Nerve Branch	V2	32 (74.4%)	6 (85.7%)	38 (76.0%)	0.52 (1)	0.470
	V3	11 (25.6%)	1 (14.3%)	12 (24.0%)		

Nerve branch comparison was limited to V2 vs. V3, as patients with V1 involvement were excluded.

## Discussion

Our prospective study adds to the expanding evidence supporting percutaneous RFR as a safe and effective intervention for drug-resistant TN. The significant reduction in pain scores from 8.04 at baseline to 2.58 at six months aligns with findings by Nanjappa et al., who reported “excellent” or “good” pain relief in 80% of patients (15). Likewise, Cheng et al. and Guo Y et al. demonstrated consistent short- and long-term efficacy with percutaneous techniques in their extensive case series (16, 22). Notably, our study incorporates patient-reported satisfaction using the PGIC scale, offering a broader perspective on outcomes beyond symptom reduction. The inclusion of PGIC as a secondary outcome adds meaningful value to the interpretation of VAS scores. While VAS quantifies pain intensity, PGIC reflects the patient's subjective global assessment of improvement, encompassing pain relief, functional ability, emotional well-being, and overall satisfaction. This dual metric approach offers a more patient-centred evaluation and aligns with current recommendations for shared decision-making in chronic pain interventions (23, 24).

Notably, our use of the PGIC scale captures not only symptomatic relief but also the patient's perceived return to functionality and satisfaction with care. This is particularly relevant in chronic pain conditions like TN, where the psychosocial impact is often substantial. Our findings of sustained satisfaction at six months reinforce the role of RFR not only as an effective analgesic intervention but also as a quality-of-life-enhancing procedure.

Our complication profile, primarily consisting of transient facial numbness (32%) and mild motor symptoms (14%), was consistent with findings reported in earlier studies (15, 25). No permanent or vision-threatening complications, such as corneal anaesthesia or anaesthesia dolorosa, were observed, reinforcing the safety of lesioning at 75°C for 60 s. *Post hoc* analysis did not reveal a significant impact of postoperative complications on satisfaction scores, although subtle trends suggest that residual sensory deficits may influence perceived outcomes. These nuances warrant further exploration in larger cohorts.

Literature comparing complications across surgical options confirms RFR's favourable safety, particularly when compared to balloon compression or microvascular decompression (MVD). Although early studies reported higher recurrence with MVD, recent systematic reviews indicate lower recurrence rates of ∼10%–15% at 2–5 years in well-selected patients (13, 14, 25), suggesting a durable benefit when feasible. Our six-month recurrence rate for RFR is promising in this context but requires longer-term follow-up (12, 26).

In comparison to conventional treatments, RFR offers a compelling balance of efficacy and safety. Pharmacological approaches such as carbamazepine and oxcarbazepine show early success rates ranging from 60%–80%, but their long-term use is often limited by adverse effects or diminishing response (7, 10). MVD, while more definitive, carries a higher surgical risk and is not suitable for all patients. Studies evaluating RFR report success rates between 75%–90% in terms of initial pain relief, with outcomes comparable to those of MVD, especially in the short to intermediate term (15, 16). These data reinforce the value of RFR as a minimally invasive yet highly effective option in the therapeutic algorithm for trigeminal neuralgia.

There was no significant correlation between demographic factors (e.g., gender, side of pain, nerve division) and procedural success, similar to earlier findings by Yadav et al. and Ravina et al. (12, 26). These results suggest RFR may be consistently effective across diverse patient profiles. However, the absence of significant subgroup differences could also reflect the study's limited power to detect small effect sizes due to the modest sample size. Larger, stratified studies are needed to confirm the universality of benefit. While our study observed a recurrence rate of just 6% within six months, extended follow-up remains crucial. Although earlier reports cited recurrence rates of 20%–30% following MVD or repeat RFR, more recent literature shows that MVD, when performed in expert centres, maintains durable relief in 80%–90% of patients (9, 14). These findings support MVD's long-term efficacy while reinforcing the importance of continued surveillance after any intervention (12, 13, 27), emphasising the importance of continued surveillance and tailored treatment planning. Our extended subset data from patients followed up to 9–12 months reinforces this need, as the majority maintained clinical improvements, although a small portion required therapeutic adjustments.

In addition to the interventional alternatives, such as sphenopalatine ganglion radiofrequency therapy (RFT), greater palatine blocks or peripheral neurectomy, were occasionally considered but remained limited in scope and durability (4, 12). While postherpetic neuralgia (PHN) may present with facial pain, its underlying pathophysiology differs substantially from classical TN and lies outside the clinical indications for RFR (9, 28, 29). Hence, our results validate RFR as a reliable and well-tolerated procedure for TN, achieving significant pain relief and high patient satisfaction with a favourable safety profile. These findings are particularly relevant in clinical settings where more invasive options, such as MVD, are contraindicated or declined. Future studies should explore predictors of long-term outcomes and the integration of patient-centred metrics in comparative treatment algorithms.

To advance clinical practice, future research should consider the development of multicenter registries tracking long-term outcomes of RFR and conduct randomised comparisons with alternative percutaneous techniques such as balloon compression, particularly in resource-limited settings. The lack of significant associations between demographic variables and treatment outcomes suggests that RFR's efficacy is broadly applicable across different patient subgroups. This supports the use of RFR as a versatile treatment option for TN.

### Limitation

While our study provides valuable insights, certain limitations must be acknowledged. The follow-up period was primarily limited to six months; however, preliminary data from 18 patients with extended follow-up up to 12 months showed promising sustained outcomes. Longer-term studies with more complete datasets are still necessary to assess the durability of pain relief and patient satisfaction on a broader scale. Additionally, the sample size, though adequate for preliminary conclusions, may limit the generalisability of the findings. Future research with larger cohorts and extended follow-up periods is warranted.

## Conclusion

Our study supports the effectiveness and safety of percutaneous RFA in treating medically refractory TN, demonstrating significant pain reduction and high patient satisfaction. With its favourable safety profile, RFA remains a valuable option within multidisciplinary TN management. Future studies should focus on refining procedural techniques and assessing long-term results to further improve clinical outcomes.

## Data Availability

The raw data supporting the conclusions of this article will be made available by the authors, without undue reservation.
